# Inactivation of H^+^-ATPase Participates in the Influence of Variation Potential on Photosynthesis and Respiration in Peas

**DOI:** 10.3390/plants9111585

**Published:** 2020-11-16

**Authors:** Lyubov Yudina, Oksana Sherstneva, Ekaterina Sukhova, Marina Grinberg, Sergey Mysyagin, Vladimir Vodeneev, Vladimir Sukhov

**Affiliations:** Department of Biophysics, N.I. Lobachevsky State University of Nizhny Novgorod, 603950 Nizhny Novgorod, Russia; lyubovsurova@mail.ru (L.Y.); sherstneva-oksana@yandex.ru (O.S.); n.catherine@inbox.ru (E.S.); mag1355@yandex.ru (M.G.); rollthebones@mail.ru (S.M.); v.vodeneev@mail.ru (V.V.)

**Keywords:** electrical signals, variation potential, systemic physiological response, H^+^-ATPase, photosynthesis, respiration, pea, protoplasts

## Abstract

Local damage (e.g., burning, heating, or crushing) causes the generation and propagation of a variation potential (VP), which is a unique electrical signal in higher plants. A VP influences numerous physiological processes, with photosynthesis and respiration being important targets. VP generation is based on transient inactivation of H^+^-ATPase in plasma membrane. In this work, we investigated the participation of this inactivation in the development of VP-induced photosynthetic and respiratory responses. Two- to three-week-old pea seedlings (*Pisum sativum* L.) and their protoplasts were investigated. Photosynthesis and respiration in intact seedlings were measured using a GFS-3000 gas analyzer, Dual-PAM-100 Pulse-Amplitude-Modulation (PAM)-fluorometer, and a Dual-PAM gas-exchange Cuvette 3010-Dual. Electrical activity was measured using extracellular electrodes. The parameters of photosynthetic light reactions in protoplasts were measured using the Dual-PAM-100; photosynthesis- and respiration-related changes in O_2_ exchange rate were measured using an Oxygraph Plus System. We found that preliminary changes in the activity of H^+^-ATPase in the plasma membrane (its inactivation by sodium orthovanadate or activation by fusicoccin) influenced the amplitudes and magnitudes of VP-induced photosynthetic and respiratory responses in intact seedlings. Decreases in H^+^-ATPase activity (sodium orthovanadate treatment) induced fast decreases in photosynthetic activity and increases in respiration in protoplasts. Thus, our results support the effect of H^+^-ATPase inactivation on VP-induced photosynthetic and respiratory responses.

## 1. Introduction

The systemic adaptation response of plants to the local action of stressors requires generation and propagation of long-distance stress signals including electrical signals. There are three main types of electrical signals, i.e., action potential, system potential, and variation potential (VP) [1,2,3,4,5,6,7,8]. Action potential is a short-term change in the electrical potential difference across the plasma membrane including depolarization followed by repolarization; the signal is induced by non-damage stressors (cooling, touch, salts, etc.) and actively propagates through the plant body [1,2]. The mechanisms of action potential are based on both transient activation of Ca^2+^, anion, and K^+^ channels [1,9] and inactivation of H^+^-ATPase [6,10] in the plasma membrane of plant cells. System potential is long-term propagating hyperpolarization [11,12], which is possibly related to the transient activation of H^+^-ATPase [11] and changes in the activity of Ca^2+^ and K^+^ channels [8,13]. VP is a long-term electrical response induced by local damage which has irregular dynamics (long-term depolarization, long-term depolarization + action potential-like spikes, or fast depolarization + slow repolarization only are possible) [1,2,3,4,5,6,7,8]. Activation of Ca^2+^ channels and transient calcium-dependent inactivation of H^+^-ATPase are probably the main mechanisms of VP [4,5,6]; however, activation of anion and K^+^ channels can also participate in the generation of the response. VP could be a local electrical response that is induced by propagation of hydraulic or chemical signals (or combinations of these signals) after plant damage [4,14,15,16].

Electrical signals can strongly influence the physiological processes in plants [2,3,4,5,6,7,8]; in particular, they stimulate expression of defense genes (e.g., genes of the proteinase inhibitor and anti-insect vegetative storage protein 2) [17,18,19,20], production of stress phytohormones (e.g., abscisic and jasmonic acids) [21,22,23,24,25,26], respiration [27,28,29], and ATP production [30]. In contrast, electrical signals suppress sieve element uploading [31], phloem mass flow [32,33], and plant growth processes [2,3,34]. Electrical signals influence transpiration [35,36,37] and photosynthetic processes (suppression of photosynthetic CO_2_ assimilation (A_CO2_), stimulation of non-photochemical quenching of chlorophyll fluorescence (NPQ) and cyclic electron flow and decrease of quantum yields of photosystem I (Φ_PSI_) and II (Φ_PSII_), etc.) [28,29,30,35,36,37,38,39,40,41] and both physiological changes modify the reflectance of plant leaves [42,43,44]. The final result of the systemic physiological response induced by electrical signals is likely to increase plant tolerance to stressors [5,7,8], which is supported by changes in the heat tolerance of photosynthetic machinery after the generation of electrical responses [45,46,47,48]. 

Revealing the mechanisms through which electrical signals influence physiological processes is important; these mechanisms have mostly been investigated for electrical signal-induced changes in photosynthetic processes [5,8]. The following three potential pathways of electrical signal influence on photosynthesis have been considered: Ca^2+^ flux into cytoplasm and stroma of chloroplasts [38], production of ROS [49], and changes in intra- and extracellular pH [35,39,41,50,51,52,53]. The last hypothesis has been supported by a number of results including (i) electrical signal-related pH increase in the apoplast and pH decrease in the cytoplasm, stroma, and lumen of chloroplasts [35,39,50,51,52]; (ii) the linear relationships between changes in extra- and intracellular pH and changes in photosynthetic parameters [52,53]; (iii) induction of photosynthetic inactivation in isolated chloroplasts after acidification of the incubation medium [35,39,50]; and (iv) induction of photosynthetic inactivation in leaves after artificial induction of H^+^ influx with protonofore treatment [39]. However, Ca^2+^ and ROS signals can strongly interact with H^+^ signals [54], i.e., arguments supporting participation of pH changes in the induction of electrical signal-caused photosynthetic responses do not exclude the indirect participation of Ca^2+^ and ROS in this induction. Few studies [53] have shown that pH changes are strongly linearly correlated with respiratory changes induced by electrical signals, i.e., pH changes also possibly participated in the induction of the respiratory response. 

Electrical signal-related changes in pH are thought to be mainly caused by the inactivation of H^+^-ATPase in the plasma membrane, because it is the main active proton transporter in this membrane [55,56] and changes in its activity play an important role in the generation of action potential [10], system potential [11,12], and especially VP [4]. This supposition was supported by our earlier theoretical work [57], in which we showed that inactivation of H^+^-ATPase could decrease the probability of CO_2_ transport into photosynthetic cells. However, some works [9,58] have experimentally shown that pH changes could be caused by anion efflux, which was accompanied by the generation of electrical signals. Potentially, K^+^ efflux can influence extracellular pH [57] because the apoplastic buffer can competitively bond both K^+^ and H^+^ [59]. 

Thus, the hypothesized key role of the inactivation of H^+^-ATPase in the induction of photosynthetic and respiratory responses caused by electrical signals requires further investigations. Our earlier work [60] preliminarily showed that decreases in the activity of H^+^-ATPase influenced photosynthetic CO_2_ assimilation and its response induced by VP; this study was devoted to further analyzing the participation of changes in H^+^-ATPase activity in forming VP-induced photosynthetic and respiratory responses in peas.

## 2. Results

### 2.1. Influence of Preliminary Treatment by Sodium Orthovanadate and Fusicoccin on Metabolic Component of Resting Potential

**Figure 1:**
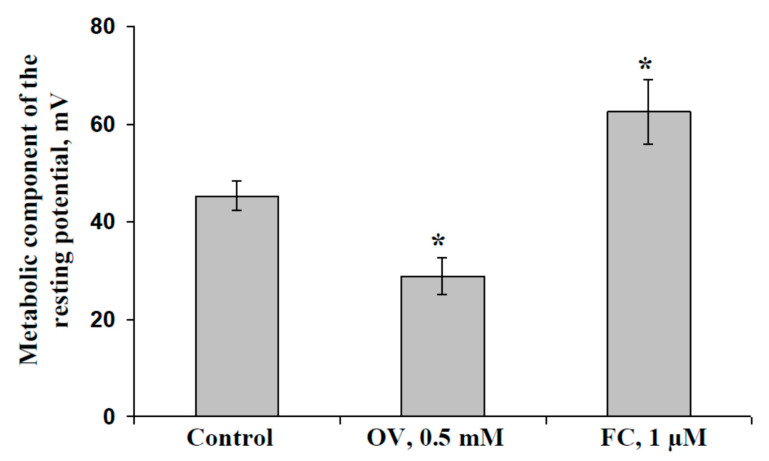
Average magnitudes of the metabolic component of the resting potential of cells of leaves in pea seedlings under control conditions and after preliminary treatment with fusicoccin (FC) and sodium orthovanadate (OV) (*n* = 6–10). The magnitude of the metabolic component, which showed H^+^-ATPase activity in the plasma membrane, was estimated on the basis of short-term changes in membrane potential after the action of the high OV concentration (see Section 4.2 and Figure S4 for details). The second mature leaves in seedlings were preliminarily treated with OV (0.5 mM) and FC (1 µM) treatments by incubation of the leaf (2 h) in solutions of these chemical agents; after that, these leaves were dried by filter paper and used for intracellular measurement of electrical activity. OV and FC were dissolved in standard solution (1 mM KCl, 0.5 mM CaCl_2_, and 0.1 mM NaCl). Similar incubation in the standard solution was used as the control. *, difference between experiment and control plants was significant (*p* < 0.05).

Figure 1 shows the influence of the preliminary treatment by 0.5 mM sodium orthovanadate (OV) and 1 µM fusicoccin (FC) on the metabolic component of the resting potential in the cells of pea seedling leaves. The magnitude of the metabolic component was used to estimate the activity of H^+^-ATPase in the plasma membrane [60,61] because this transporter is the main mechanism through which ions are actively transported in higher plants [55,56].

We found that the magnitude of the metabolic component of the resting potential was about 45 mV in control leaf cells. Preliminary treatment with OV moderately decreased this magnitude, which was about 29 mV in treated pea seedling leaves. In contrast, preliminary treatment with FC increased the magnitude of the metabolic component of the resting potential; this magnitude was about 63 mV in treated leaves and both effects were significant. The results showed that OV could be used as an inhibitor of H^+^-ATPase and FC could be used as activator of this transporter; these treatments could be used for moderate modification of H^+^-ATPase activity. 

### 2.2. Influence of Preliminary Treatment by Sodium Orthovanadate and Fusicoccin on Amplitude of Local Burning-Induced Variation Potentials

**Figure 2:**
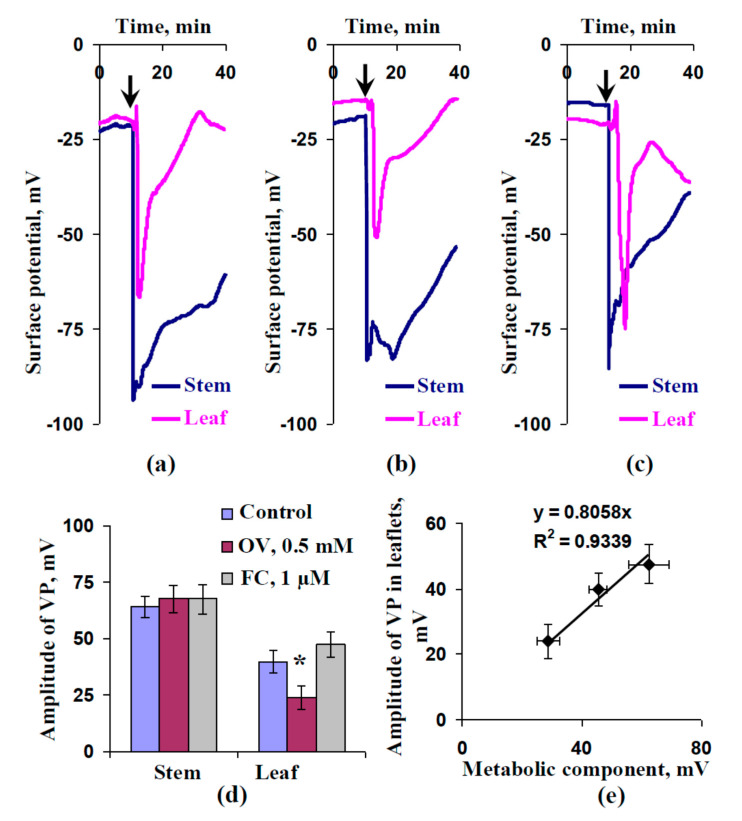
Examples of variation potential (VP) measurements in pea seedlings under control conditions (**a**) and after preliminary treatment by sodium orthovanadate (OV) (**b**) or fusicoccin (FC) (**c**); average amplitudes of VP (**d**) (*n* = 6–15) and scatter plot between values of the metabolic component and VP amplitudes in leaflets (**e**). The preliminary OV (0.5 mM) and FC (1 µM) treatment of the second mature leaves in seedlings was performed by incubation of the leaf (2 h) in solutions of these chemical agents; after that, these leaves were dried using filter paper and used for the extracellular measurement of electrical activity. OV and FC were dissolved in standard solution (1 mM KCl, 0.5 mM CaCl_2_, and 0.1 mM NaCl). Similar incubation in the standard solution was used as the control. The arrows mark the local burning of the first mature leaf (flame, 2–3 s). Extracellular measurements of surface potentials were recorded in the second leaves and in the stems near these leaves. The values of the metabolic component were taken from Figure 1. *, statistically significant difference between experiment and control plants (*p* < 0.05). R^2^, determination coefficient.

Figure 2 shows that local burning of the first mature leaf induced electrical signals, which propagated into the second mature leaf. The electrical signal had an irregular shape (including a fast peak and slow depolarization) and it was long term (tens of minutes), indicating the electrical signal could be classified as a VP [4,6]. 

The amplitudes of the variation potentials in the stem near the second leaf were similar in all investigated variants; they were about 64–68 mV (Figure 2d). This result agrees with the absence of plant treatments in these zones. In contrast, the VPs in the leaflet of the second leaf had different amplitudes. The average amplitude of the VP in the leaflets of control seedlings was about 40 mV. The OV leaf treatment significantly decreased the average VP amplitude; it was about 25 mV in treated leaves. The influence of the FC treatment was not significant; however, an increase in VP amplitude was observed. The modified amplitude of the VP after FC leaf treatment was about 50 mV. Figure 2e shows that VP amplitudes were linearly related to values of the metabolic component of the resting potential. The results showed that preliminary modification of H^+^-ATPase activity could influence VP parameters. 

### 2.3. Influence of Preliminary Treatment by Sodium Orthovanadate and Fusicoccin on Local Burning-Induced Changes in Photosynthetic Parameters 

**Figure 3:**
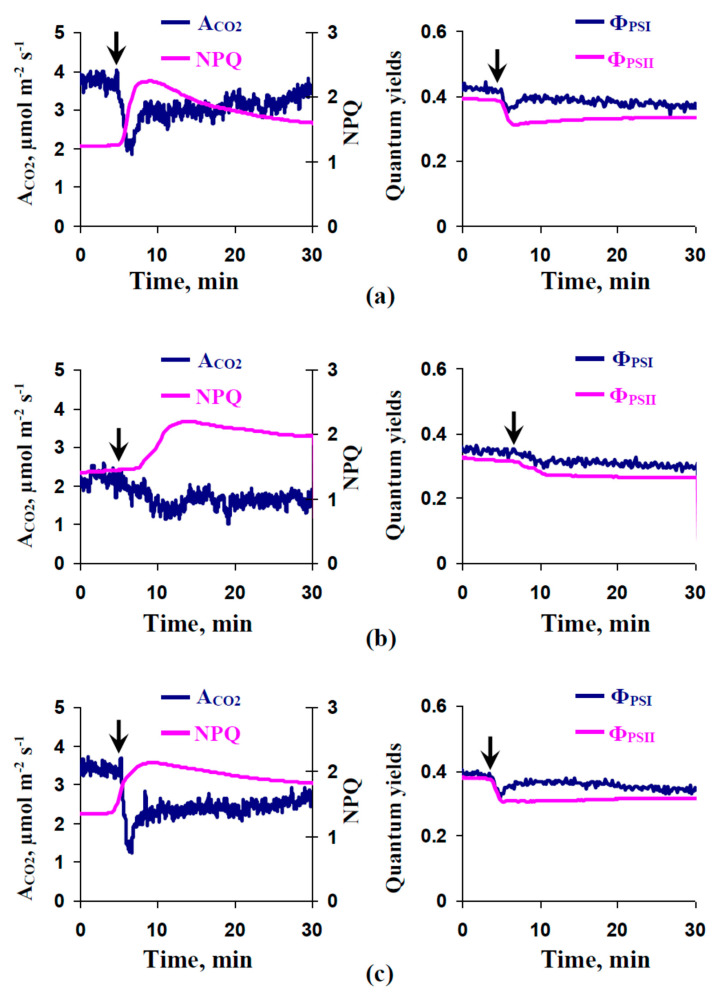
Examples of local burning-induced changes in photosynthetic CO_2_ assimilation (A_CO2_), non-photochemical quenching of chlorophyll fluorescence (NPQ), and quantum yields of photosystems I (Φ_PSI_) and II (Φ_PSII_), in the second leaf of pea seedlings under control conditions (**a**) and after preliminary treatment by sodium orthovanadate (OV) (**b**) and fusicoccin (FC) (**c**). The preliminary OV (0.5 mM) and FC (1 µM) treatments of the second mature leaves in seedlings were performed by incubation of the leaf (2 h) in solutions of these chemical agents. After that, these leaves were dried by filter paper and used for photosynthetic measurements. OV and FC were dissolved in standard solution (1 mM KCl, 0.5 mM CaCl_2_, and 0.1 mM NaCl). Similar incubation in the standard solution was used as the control. The arrows mark the local burning of the first mature leaf (flame, 2–3 s).

Figure 3a shows that local burning induced photosynthetic changes in control plants including suppression of photosynthetic CO_2_ assimilation, decreasing quantum yields of photosystems I and II, and increasing non-photochemical quenching of chlorophyll fluorescence. Figure 3b,c depicts the local burning-induced photosynthetic changes after preliminary treatment of leaves using OV and FC, respectively. The OV treatment decreased these photosynthetic changes; in contrast, FC increased the changes in A_CO2_ and weakly influenced changes in other parameters. 

The photosynthetic changes showed photosynthetic inactivation; they were typical photosynthetic responses to local burning inducing the propagation of variation potentials [5]. The correlation analysis showed (Figure S1) that magnitudes of changes of most of the investigated photosynthetic parameters (A_CO2_, Φ_PSII_, and NPQ) were significantly linearly correlated with the amplitudes of the VPs in leaflets of the second leaves. This result supports a key role of variation potentials in the induction of photosynthetic changes. 

**Figure 4:**
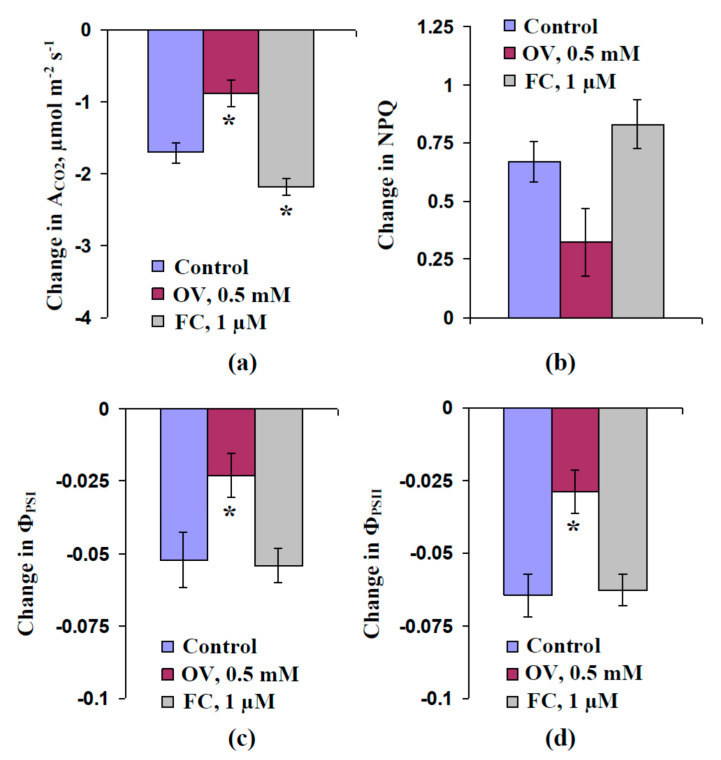
Magnitudes of local burning-induced changes in photosynthetic CO_2_ assimilation (A_CO2_) (**a**), non-photochemical quenching of chlorophyll fluorescence (NPQ) (**b**), and quantum yields of photosystems I (Φ_PSI_) (**c**) and II (Φ_PSII_) (**d**), in the second leaf of pea seedlings under control conditions and after preliminary treatment by sodium orthovanadate (OV) and fusicoccin (FC) (*n* = 5–6). The preliminary OV (0.5 mM) and FC (1 µM) treatments of the second mature leaves in seedlings were performed by incubation of the leaf (2 h) in solutions of these chemical agents. After that, these leaves were dried by filter paper and used for photosynthetic measurements. OV and FC were dissolved in standard solution (1 mM KCl, 0.5 mM CaCl_2_, and 0.1 mM NaCl). Similar incubation in the standard solution was used as the control. The first mature leaf was burned (flame, 2–3 s). *, significant difference between experiment and control plants (*p* < 0.05).

Figure 4 shows the average magnitudes of changes in the investigated photosynthetic parameters. The OV treatment significantly decreased the magnitudes of changes in A_CO2_, Φ_PSI_, and Φ_PSII_; insignificant decreases in NPQ were also observed. The FC treatment increased the magnitudes of changes in A_CO2_ (significant decrease) and NPQ (insignificant increase). In contrast, the magnitudes of decreases of Φ_PSI_ and Φ_PSII_ were approximately equaled to that of the control. 

**Figure 5:**
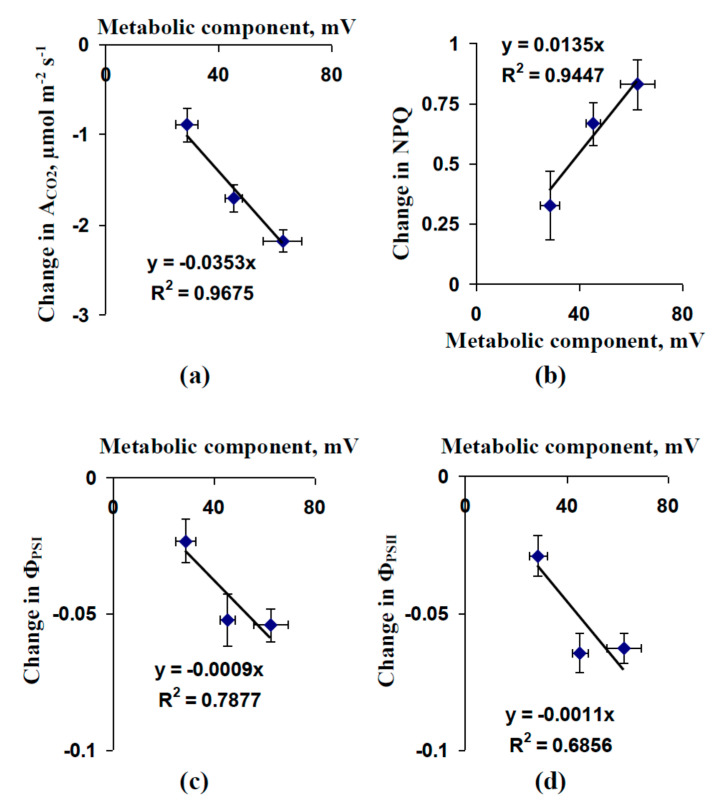
Scatter plots between the values of the metabolic component of the membrane potential and magnitudes of local burning-induced changes in photosynthetic CO_2_ assimilation (A_CO2_) (**a**), non-photochemical quenching of chlorophyll fluorescence (NPQ) (**b**), and quantum yields of photosystems I (Φ_PSI_) (**c**) and II (Φ_PSII_) (**d**), in the second leaf of pea seedlings. Data from Figure 1 and Figure 3 were used.

The scatter plots in Figure 5 compare the magnitudes of local burning-induced changes in photosynthetic parameters and the average magnitudes of the metabolic component of the resting potential in leaf cells (data from Figure 1 and Figure 4 were used). The magnitudes of the photosynthetic changes could be described as linear functions of the magnitude of the metabolic component. The determination coefficients varied from about 0.68–0.97. 

The local burning-induced changes in the photosynthetic parameters and the modifications of these changes after OV and FC treatments were not caused by changes in stomatal opening. Figure S2a shows that local burning induced changes in transpiration; however, the dynamics of this change differed from the changes in the photosynthetic parameters. The magnitudes of transpiration changes were weakly correlated with the magnitudes of the changes in the photosynthetic parameters (Figure S2b). 

### 2.4. Influence of Preliminary Treatment by Sodium Orthovanadate and Fusicoccin on Local Burning-Induced Changes in Respiration 

**Figure 6:**
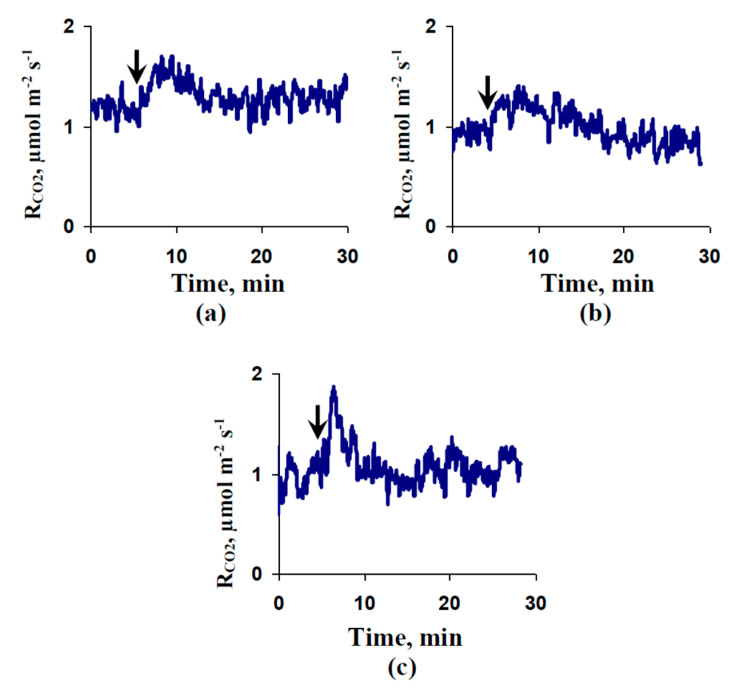
Examples of local burning-induced changes in respiration (R_CO2_) in the second leaf of pea seedlings under control conditions (**a**) and after preliminary treatment by sodium orthovanadate (OV) (**b**) and fusicoccin (FC) (**c**). Respiration was measured under dark conditions. The preliminary OV (0.5 mM) and FC (1 µM) treatments of the second mature leaves in seedlings were performed by incubation of the leaf (2 h) in solutions of these chemical agents. After that, these leaves were dried by filter paper and used for respiratory measurements. OV and FC were dissolved in standard solution (1 mM KCl, 0.5 mM CaCl_2_, and 0.1 mM NaCl). Similar incubation in the standard solution was used as a control. The arrows mark the local burning of the first mature leaf (flame, 2–3 s).

Figure 6a shows the activation of respiration that was measured under dark conditions in the second leaves after local burning of the first leaf in control seedlings. The duration of the activation was about 10 min, which was shorter than the duration of photosynthetic changes (tens of minutes). The OV treatment decreased the respiratory activation (Figure 6b). In contrast, the FC treatment weakly influenced this activation (Figure 6c). The amplitudes of the variation potentials in the leaflets of the second leaf and the magnitudes of the respiratory activation were significantly linearly correlated (Figure S3). The results supported the participation of VPs in the induction of the respiratory response after local burning.

**Figure 7:**
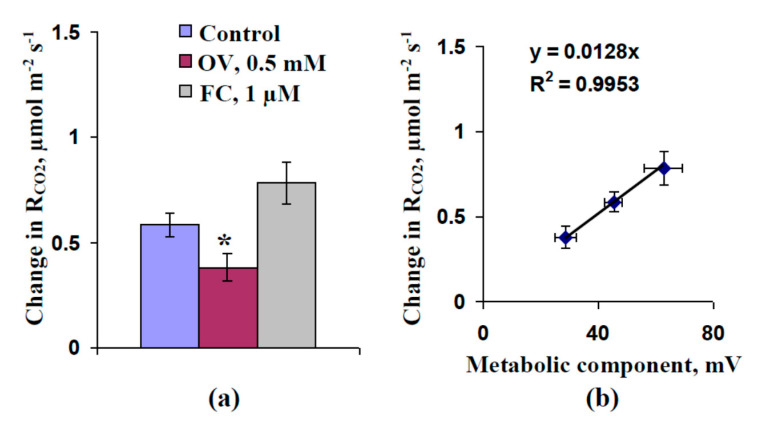
Magnitudes of local burning-induced changes in respiration (R_CO2_) (**a**) and scatter plot between the values of the metabolic component of the membrane potential and the magnitudes of these changes (**b**) (*n* = 5–6). Respiration was measured under dark conditions. The preliminary OV (0.5 mM) and FC (1 µM) treatments of the second mature leaves in seedlings were performed by incubation of the leaf (2 h) in solutions of these chemical agents. After that, these leaves were dried using filter paper and used for respiratory measurement. OV and FC were dissolved in standard solution (1 mM KCl, 0.5 mM CaCl_2_, and 0.1 mM NaCl). Similar incubation in the standard solution was used as a control. The first mature leaf was burned (flame, 2–3 s). The values of the metabolic component were taken from Figure 1. *, significant difference between experiment and control plants (*p* < 0.05).

Figure 7a shows the average magnitudes of local burning-induced respiratory activations. The control magnitude of the respiratory response was about 0.6 µmol m^−2^ s^−1^. The preliminary OV treatment significantly decreased the average magnitude of the respiratory response (about 0.4 µmol m^−2^ s^−1^). In contrast, the preliminary FC treatment increased this magnitude (about 0.8 µmol m^−2^ s^−1^); however, the increase was not significant. 

Figure 7b shows scatter plots between the magnitudes of the metabolic component of the resting potential in leaf cells and those of the respiratory activations induced by local burnings. We found that the dependence of the respiratory activation magnitude on the magnitude of the metabolic component could be accurately described by a linear equation (R^2^ > 0.99).

### 2.5. Influence of Injection of Sodium Orthovanadate on Photosynthesis and Respiration of Protoplasts 

Protoplasts, which were isolated from leaf of pea seedlings, were used for additional analysis of the participation of H^+^-ATPase in the plasma membrane on the induction of photosynthetic and respiratory responses after local damage and propagation of VP. 

**Figure 8:**
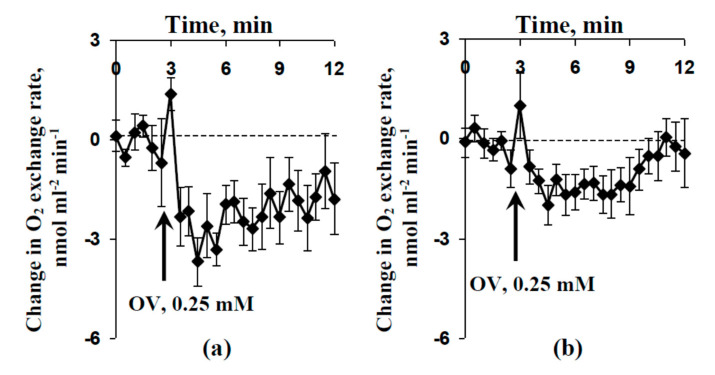
Average changes in O_2_ exchange rate induced by injection of sodium orthovanadate (OV) in pea protoplasts under light (**a**) and dark (**b**) conditions (*n* = 7–10). OV was dissolved in standard solution (1 mM KCl, 0.5 mM CaCl_2_, and 0.1 mM NaCl); the arrows mark the OV injection into the protoplast incubation medium (injection volume was 30 µL and final OV concentration was 0.25 mM). The incubation medium for protoplasts included sorbitol (400 mM), CaCl_2_ (5 mM), MgCl_2_·6H_2_O (5 mM), NaCl (20 mM), and MES-KOH (30 mM). The pH was about 5.5. The final volume (the incubation medium + protoplasts) was 1.2 mL. Blue actinic light (460 nm, 240 µmol m^−2^ s^−1^) was used in the experiment under light conditions. In each experiment, the change in O_2_ exchange rate was calculated as the difference between the O_2_ release/consumption rate in the variant with injection of OV (experiment) and the rate in the variant with injection of standard solution (control). Increases in O_2_ exchange rate indicated the increase in O_2_ release (under light conditions) or decrease in O_2_ consumption (under dark conditions). The decrease in O_2_ exchange rate indicated a decrease in O_2_ release (under light conditions) or increase in O_2_ consumption (under dark conditions).

The influence of the OV injection (final concentration of sodium orthovanadate was 0.25 mM) on the O_2_ exchange rate of protoplasts was investigated. Figure 8a shows that this injection induced a fast decrease in the O_2_ exchange rate under light conditions (under illumination by blue actinic light), which showed a decrease in O_2_ release. This decrease seemed to be related to the inactivation of photosynthetic processes; the effect was similar to photosynthetic inactivation after local burning and propagation of the VP. Figure 8b shows that the OV injection also induced a fast decrease in the O_2_ exchange rate under dark conditions, shown by the increase in O_2_ consumption, meaning this effect showed respiratory activation.

The magnitude of the decrease in the O_2_ exchange rate in protoplasts induced by the OV injection under light conditions (Figure 8a) was greater than that induced under dark conditions (Figure 8b). The result agreed with the large magnitude of the local burning-induced decrease in A_CO2_ (Figure 4a) and the small magnitude of the local burning-induced increase in R_CO2_ (Figure 7a).

**Figure 9:**
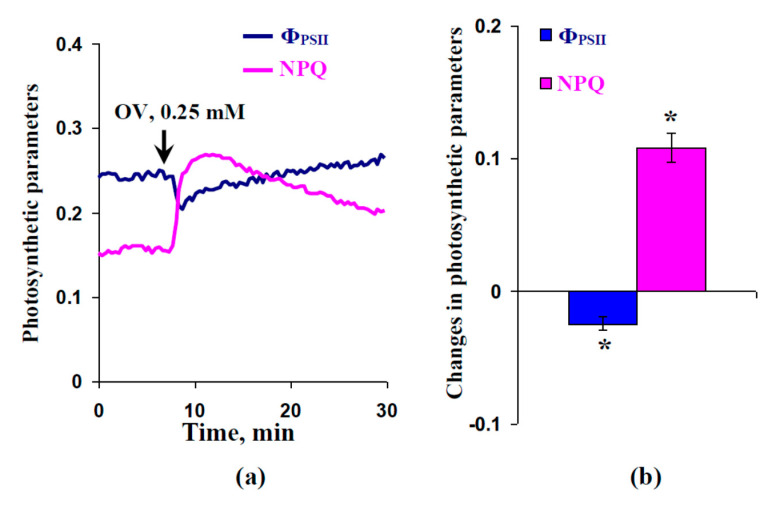
Example of changes in quantum yield of photosystems II (Φ_PSII_) and non-photochemical quenching of chlorophyll fluorescence (NPQ) after injection of sodium orthovanadate (OV) (**a**) and average magnitudes of these changes (**b**) in protoplasts of peas (*n* = 6). OV was dissolved in standard solution (1 mM KCl, 0.5 mM CaCl_2_, and 0.1 mM NaCl); the arrows mark the OV injection into the protoplast incubation medium (injection volume was 30 µL and final OV concentration was 0.25 mM). The incubation medium for protoplasts included sorbitol (400 mM), CaCl_2_ (5 mM), MgCl_2_·6H_2_O (5 mM), NaCl (20 mM), and MES-KOH (30 mM). The pH was about 5.5. The final volume (the incubation medium + protoplasts) was about 3 mL. Photosynthetic measurements were recorded under blue actinic light (460 nm, 108 µmol m^−2^ s^−1^). *, the average change was significant (*p* < 0.05).

The influence of the OV injection (final concentration of sodium orthovanadate was 0.25 mM) on the parameters of photosynthetic light reactions in protoplasts was also investigated (Figure 9). We found that the OV injection induced a fast decrease in the quantum yield of photosystem II and increased non-photochemical quenching; these changes were significant. The dynamics of the photosynthetic changes were similar to the dynamics of these changes after local burning and VP propagation.

## 3. Discussion

Variation potential plays an important role in inducing systemic plant adaptation response to local damage [8]. In particular, photosynthesis and respiration are key targets of the influence of VP. A VP can suppress photosynthetic processes, and thereby modify the tolerance of photosynthetic machinery [45,46,47], meaning it participates in the plant adaptation to stressors [5,8]. A VP also activates respiration in plant leaves [29,30]. Both changes increase the ATP content in leaves [30]. This increase probably participates in plant repair after the actions of a stressor [5,8,62,63]. 

The increase in pH in the apoplast and the decrease in pH in the cytoplasm, stroma, and lumen of the chloroplasts are probably the main mechanisms through which the photosynthetic response is induced after VP propagation [35,39,50,51,52]. Alternative mechanisms also probably induce the photosynthetic response (Ca^2+^ influx [38] or stimulation of ROS production [49]); however, their processes can strongly interact with changes in pH [8]. VP-induced changes in respiration, also being induced by pH changes, cannot be excluded, as our earlier results showed that the dynamics of the respiration response is linearly related to the dynamics of pH increase in the apoplast [52]. 

The transient inactivation of H^+^-ATPase in the plasma membrane, which is the main action of VPs [1,2,3,4,5,6], is a potential reason for these pH changes [39,51,60]. However, alternative mechanisms of pH changes, including anion [9,58] and K^+^ [57] effluxes, are also probable. Our results experimentally support the key role of H^+^-ATPase inactivation in the induction of VP-caused photosynthetic and respiratory responses. First, we showed that the preliminary modification of H^+^-ATPase activity (preliminary OV and FC treatment) influenced the parameters of VP and photosynthesis and respiration responses, its inactivation decreased the amplitude of electrical signals (Figure 3) and magnitudes of photosynthetic (Figure 4) and respiratory (Figure 7) changes. In contrast, its activation can increase the magnitude of VP-induced changes in A_CO2_. Second, the VP amplitudes and magnitudes of photosynthetic and respiratory responses are linearly related to the magnitudes of the metabolic component of the resting potential, which demonstrates the activity of H^+^-ATPase [60,61] (Figure 2e, Figure 5 and Figure 7b). Third, the injection of a H^+^-ATPase inhibitor (OV) into a suspension of pea protoplasts induced photosynthetic and respiratory changes (Figure 8 and Figure 9) that were similar to changes induced by VP. 

Notably, OV can also influence Ca^2+^-ATPase in the plasma membrane; participation of Ca^2+^-ATPase inactivation in the identified effects cannot be fully excluded (maybe as a result of disturbing Ca^2+^ signaling, which plays an important role in VP generation [16]). However, the revealed linear relationships between the magnitudes of the investigated responses (VP, photosynthetic and respiratory changes) and the magnitudes of the metabolic component of the resting potential (which are mainly caused by H^+^-ATPase activity [55,56]) do not support this possibility. Additionally, calcium signaling is mainly based on the strong activation of Ca^2+^ channels [16]; Ca^2+^-ATPase-inactivation-induced changes in the Ca^2+^ concentration should be rather slow. However, our experiments using protoplasts showed fast changes in the photosynthetic and respiratory parameters after OV injection. 

**Figure 10:**
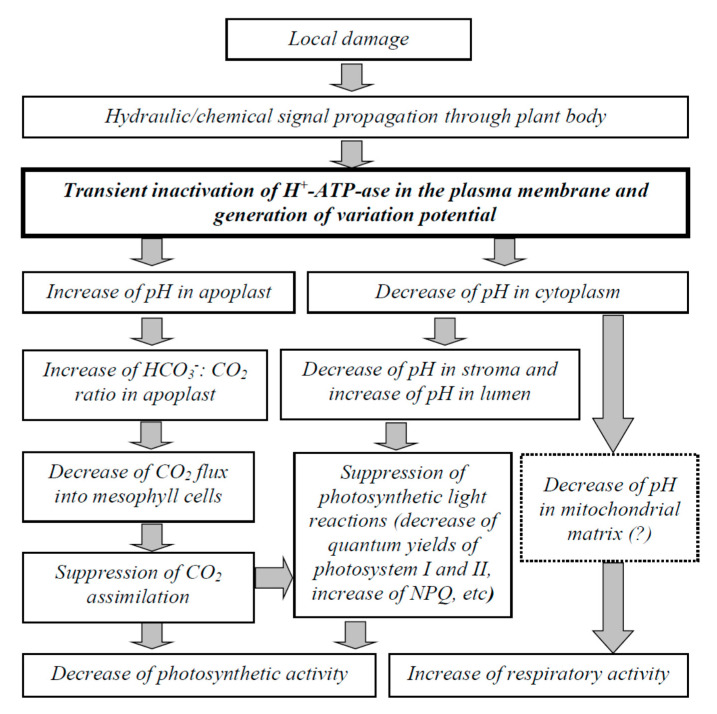
Potential pathways through which the variation potential-related inactivation of H^+^-ATPase in the plasma membrane influences photosynthesis and respiration. It is hypothesized that local damage induces the propagation of hydraulic and (or) chemical signals, which decrease the activity of H^+^-ATPase in the plasma membrane (maybe through Ca^2+^ flux into the cytoplasm), and thereby generate variation potential. Inactivation of H^+^-ATPase both increases pH in the apoplast and decreases pH in the cytoplasm. The increased apoplastic pH increases the HCO_3_^−^/CO_2_ ratio in the apoplast and decreases the CO_2_ flux into the mesophyll cells through the plasma membrane because CO_2_ is more permeable through biological membranes than HCO_3_^−^. The decreased CO_2_ flux induces the suppression of photosynthetic CO_2_ assimilation. The decreased pH in the cytoplasm can decrease the pH in the stroma and lumen of chloroplasts; suppression of photosynthetic light reactions (decrease in quantum yields of photosystem I and II, increase in NPQ, etc.) is induced by these changes in pH. In contrast, the decreased pH in the cytoplasm can stimulate respiration. The effect is probably caused by the decrease in pH in the mitochondrial matrix and the stimulation of respiratory electron flow. Thus, both the decrease in photosynthetic activity and the increase in respiration are the results of the propagation of variation potentials.

Figure 10 shows that the potential pathways through H^+^-ATPase inactivation participates in the induction of the photosynthetic and respiratory responses by VP. Local damage induces the propagation of hydraulic or chemical signals (or a combination of these signals) through the plant body [1,2,3,4,14,15]. Propagating signals induce the transient inactivation of H^+^-ATPase in the plasma membrane [2,4,5,6,25], which is probably caused by the Ca^2+^ flux into the cells and the increase of calcium concentration in the cytoplasm. 

The two main possible influences of H^+^-ATPase inactivation on photosynthetic processes are [5,8] an increase in apoplastic pH and a decrease in intracellular pH. Increased pH in the apoplast can increase the ratio of HCO_3_^−^ concentration to CO_2_ concentration in the apoplast [50,57,64]. Considering the low permeability of the biological membranes for HCO_3_^−^ [64,65], this process should decrease the CO_2_ conductance through the mesophyll cells and suppress A_CO2_ [57,60,61]. An alternative influence of pH changes on the CO_2_ influx is related to the suppression of CO_2_ transport through aquaporins [5,36]; however, this hypothesis requires further experimental investigations. 

H^+^-ATPase inactivation-related acidification of the cytoplasm [35,39,50,52], which causes decreased pH in the stroma and lumen of chloroplasts [51], can also induce photosynthetic inactivation [5,8]. In particular, lumen acidification can induce increased NPQ [66,67] and decreased photosynthetic electron flow through photosystem II [68,69,70]. Stroma acidification induces ferredoxin-NADP^+^ reductase accumulation at the thylakoids in the Tic62 and TROL complexes [71,72], which also suppress photosynthetic light reactions. 

The pathways through which VP-related H^+^-ATPase inactivation influences respiration require further investigations. The pH of the incubation medium is known to influence the respiratory rate in mitochondria [73,74,75,76]. In particular, acidification of the incubation medium to a pH of about 6.5 [75,76] or 7 [74] can activate respiration. The pH in the matrix of the mitochondria is related to the pH in the cytoplasm [77,78]; thus, we speculate that VP-caused acidification of the cytoplasm [35,39], which is the result of H^+^-ATPase inactivation, should decrease the matrix pH. Potentially, matrix acidification can stimulate respiration as a result of the activation of the mitochondrial electron transport chain by increased concentration of H^+^ or changes in the activity of alternative oxidase [75,76]. The first pathway is supported by our earlier results, which showed that the VP-induced increase of ATP content was accompanied by respiratory activation [30]; however, this finding requires future analysis.

Thus, we showed that the VP-related inactivation of H^+^-ATPase plays an important role in the induction of photosynthetic inactivation and respiratory activation. Both responses probably participate in the increase in plant tolerance to stressors and the stimulation of repair processes (maybe as a result of the increase in the ATP content in plants) [5,8]. 

## 4. Materials and Methods 

### 4.1. Plant Materials and Preliminary Tretmants

We investigated 2–3-week-old pea seedlings (*Pisum sativum* L.) that were hydroponically cultivated (a half-strength Hoagland–Arnon medium) in a Binder KBW 240 climatic chamber (Binder GmbH, Tuttlingen, Germany) at 23 °C under a 16/8 light/dark photoperiod. White light was used (about 170 µmol m^−2^ s^−1^; Fluora^®^ growth lamps, Binder GmbH, Tuttlingen, Germany).

For modification of the initial activity of H^+^-ATPase in the plasma membrane, the 2nd mature leaves of seedlings were preliminarily treated by 0.5 mM sodium orthovanadate (OV; Sigma-Aldrich, St. Louis, MO, USA) or 1 µM fusicoccin (FC; Sigma-Aldrich, St. Louis, MO, USA). OV was used as the inhibitor of H^+^-ATPase in the plasma membrane [46,60] because this agent has been widely used for the suppression of P-type transport ATPases (including H^+^-ATPase [11]). FC was used as the activator of H^+^-ATPase [11]. In accordance with previous works [11,46,60], the preliminary OV (0.5 mM) and FC (1 µM) treatments were performed by incubation of the leaf (2 h) in solutions of these chemical agents. After that, these leaves were dried using filter paper and used for the next measurements. OV and FC were dissolved in standard solution (1 mM KCl, 0.5 mM CaCl_2_, and 0.1 mM NaCl). A similar treatment with the standard solution was used as the control.

### 4.2. Estimation of the Metabolic Component of the Resting Potential

The magnitude of the metabolic component of the resting potential was used for an estimation of H^+^-ATPase activity in the plasma membrane [60,61] because this transporter plays a key role in the active transport of ions in higher plants [55,56]. Measurements were performed in the mesophyll cells of the second mature leaves. 

The intracellular plasma membrane potential was measured using a patch clamp system on the basis of a SliceScope Pro 2000 microscope (Scientifica, Uckfield, UK), which included a MultiClamp 700B amplifier (Molecular Devices, San Jose, CA, USA), a DIGIDATA 1550 data acquisition system (Molecular Devices), and a personal computer. Micropipettes were fabricated on a P-97 Sutter Micropipette Puller (Sutter Instrument, Novato, CA, USA) with a tip diameter of about 1 µm and a resistance of about 40 MOhm. The reference electrode (chlorinated silver wire) was immersed in the solution in the experimental chamber. The measured potential was stable for 10 min of the experiment after electrode injection (total duration of experiment). The average value of the initial resting potential in control plants was −127 mV. The standard error was about 6 mV. 

In accordance with our earlier works [60,61], we measured the metabolic component on the basis of the amplitude of the fast depolarization of the membrane potential (several minutes) after the injection of OV into the solution in the experimental chamber (Figure S4). The final concentration of OV was 5 mM. This concentration fully suppressed H^+^-ATPase activity. The total duration of the potential measurement after the OV injection was about 7 min. 

### 4.3. Local Burning of Seedlings and Measurement of Electrical Signals

**Figure 11:**
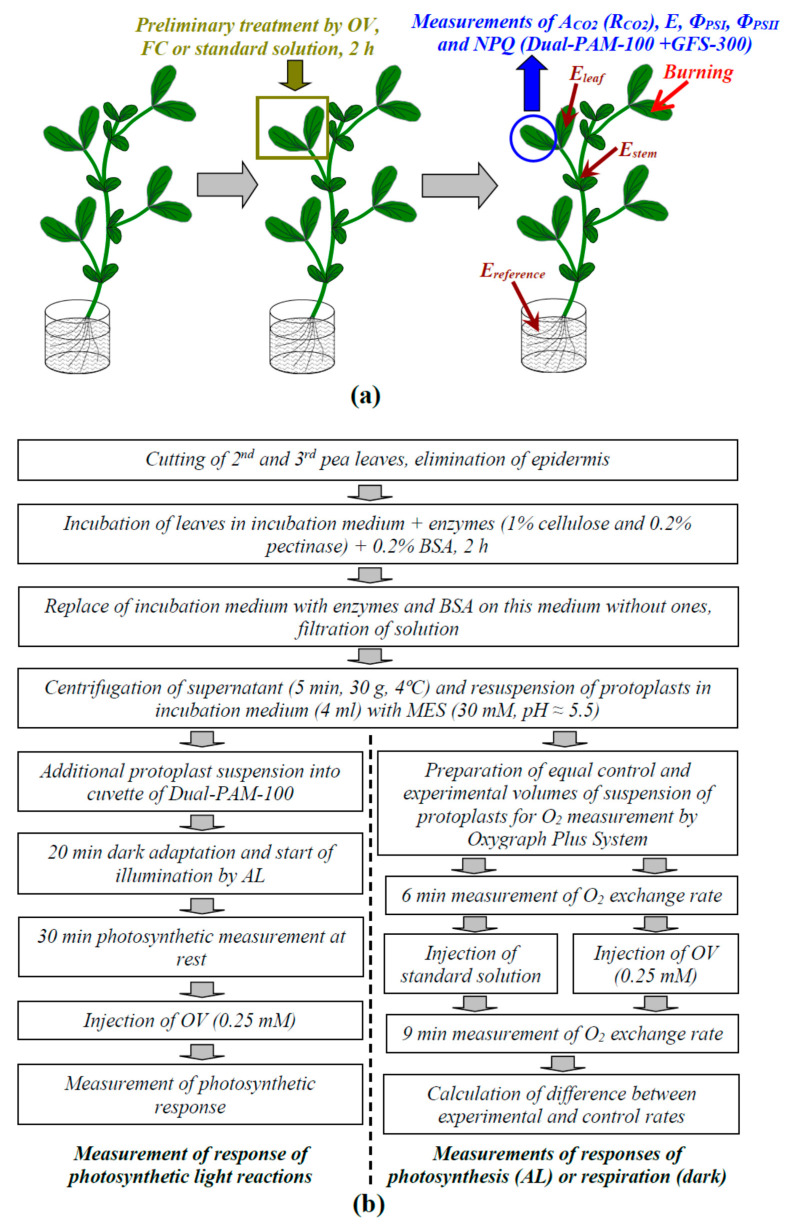
(**a**) Schema of investigations of the influence of the preliminary modification of the activity of H^+^-ATPase in the plasma membrane on electrical signal-induced photosynthetic and respiratory responses. Activity of H^+^-ATPase was decreased by leaf treatment with sodium orthovanadate (OV) and increased by treatment with fusicoccin (FC). The preliminary OV (0.5 mM) and FC (1 µM) treatments of the second mature leaves in seedlings was performed by incubation of the leaf (2 h) in solutions of these chemical agents. After that, these leaves were dried using filter paper and used for extracellular measurement of photosynthetic and electrical activities. OV and FC were dissolved in standard solution (1 mM KCl, 0.5 mM CaCl_2_, and 0.1 mM NaCl). Similar treatment using the standard solution was used as the control. The arrows mark the local burning of the first mature leaf (flame, 2–3 s). E_leaf_ and E_stem_ are the measuring electrodes that were placed on the second leaves (center of leaflet) and stems near these leaves, respectively; E_reference_ is the reference electrode. (**b**) Schema of experiments using a suspension of protoplasts from pea leaves. Photosynthetic light reactions were investigated under actinic light (AL, 460 nm, 239 µmol m^−2^ s^−1^) using a Dual-PAM-100. O_2_ exchange rate was investigated under AL (for photosynthetic investigations) or under dark conditions (for respiratory investigations) using an Oxygraph Plus System. The standard solution included 1 mM KCl, 0.5 mM CaCl_2_, and 0.1 mM NaCl.

Local burning of the upper part of the first mature leaf of pea seedlings (Figure 11a) was induced by an open flame (3–4 s, about 1 cm^2^) in accordance with our previous works [39,40,41,42,43,44,45,46,47]. This damage is the standard stimulus for induction of VPs [4]. The induction of a VP occurred 1.5 h after the fixation of seedlings in the measuring system.

The propagation of electrical signals into the second mature leaf was measured on the basis of changes in the surface electrical potential using extracellular measurements. The method was simple and suitable for the measurement of the electrical responses in different points of the plants [16,41] and for simultaneous measurement of the photosynthetic and respiratory parameters [39]. The surface electrical potential was measured using Ag^+^/AgCl electrodes (RUE Gomel Measuring Equipment Plant, Gomel, Belarus), a high-impedance IPL-113 amplifier (Semico, Novosibirsk, Russia), and a personal computer. The electrodes were connected to the center of the leaflet of the investigated leaf (E_leaf_) and to the stem near this leaf (E_stem_). Measuring electrodes were contacted with the stem and leaflet of the second leaf via Uniagel conductive gel (Geltek-Medica, Moscow, Russia), according to our previous studies [39,40]. The reference electrode (E_reference_) was placed in a solution surrounding the root. Different leaves were used for control measurements and for measurements in leaves after OV and FC treatments. 

### 4.4. Measurements of Photosynthetic and Respiratory Responses in Intact Leaves

A GFS-3000 gas analyzer (Heinz Walz GmbH, Effeltrich, Germany), Dual-PAM-100 Pulse-Amplitude-Modulation (PAM)-fluorometer (Heinz Walz GmbH, Effeltrich, Germany), and Dual-PAM gas-exchange Cuvette 3010-Dual common measuring head (Heinz Walz GmbH, Effeltrich, Germany) were used for photosynthetic investigations (Figure 11a). The concentration of CO_2_ in the measuring cuvette was 360 ppm, relative humidity was about 70%, and temperature was 23 °C. The pulses of measuring blue light (460 nm, 24 µmol m^−2^ s^−1^, 2.5 µs), red saturation pulses (630 nm, 300 ms, 10,000 µmol m^−2^ s^−1^), and blue actinic light (AL, 460 nm, 240 µmol m^−2^ s^−1^) were used for photosynthetic analysis. 

The measurements of photosynthetic parameters started after a 20 min dark interval. First, the initial and maximum levels of photosystem II fluorescence (*F*_0_ and *F_m_*, respectively) and maximum light absorption by photosystem I (*P_m_*) were measured. Leaf illumination by AL and generation of saturation pulses were started after that; the interval between saturation pulses was 10 s. The current levels of fluorescence (*F*), maximum fluorescence level after the preliminary illumination (*F_m_*’), current light absorption by photosystem I (*P*), and maximum light absorption by photosystem I after the preliminary illumination (*P_m_’*) were measured for every saturation pulse. The parameters of the photosynthetic light reactions, including Φ_PSI_, Φ_PSII_, and NPQ, were calculated on the basis of the measured parameters in accordance with standard equations [66,67,79]. GFS-3000 (Heinz Walz GmbH, Effeltrich, Germany) was used for measurements of A_CO2_ and E, which were automatically calculated by GFS-3000 software. 

Only GFS-3000 (Heinz Walz GmbH, Effeltrich, Germany) was used for respiratory measurements. R_CO2_ was calculated on the basis of the CO_2_ assimilation rate, which was measured under dark conditions ($R_{\mathrm{CO}2}=-A_{\mathrm{CO}2}$).

### 4.5. Preparation of Protoplasts from Pea Leaves and Measurements of Their Photosynthetic and Respiratory Parameters

Protoplasts, which were isolated from pea leaves, were used for additional analysis of the participation of the plasma membrane H^+^-ATPase in the photosynthetic and respiratory responses induced by electrical signals. A schema of protoplasts isolation and further experiments is briefly shown in Figure 11b. OV was injected to imitate the fast H^+^-ATPase inactivation that accompanies VP. 

The 2nd and 3rd mature pea leaves (about 25) were cut off and the epidermis was eliminated using a razor blade. These leaves were placed in the incubation medium (400 mM sorbitol, 5 mM CaCl_2_, 5 mM MgCl_2_·6H_2_O, 20 mM NaCl, 30 mM MES-KOH (pH = 5.5)) + enzymes (1% cellulose and 0.2% pectinase) + 0.2% bovine serum albumin (BSA) for 2 h under light white light (about 42 µmol m^−2^ s^−1^) and controlled temperature (28 °C). The manufacturer of all reagents was Sigma-Aldrich (St. Louis, MO, USA). After that, the solution was replaced on the incubation medium without enzymes, and BSA and protoplasts were separated from the leaves for 5 min. The resulting suspension was passed through a filter with 50 μm pores and centrifuged (5 min, 30× *g*, 4 °C). Sedimented protoplasts were resuspended in the incubation medium (4 mL).

A modification of Dual-PAM-100 for suspension analysis was used for investigation of the parameters of photosynthetic light reactions in the protoplasts. The experimental procedure was similar to that for the photosynthetic measurements in intact leaves; however, the parameters of photosystem I were not investigated because using a magnetic stirrer disturbed their measurements. The photosynthetic response was induced by injection of OV (final concentration was 0.25 mM). The injection was performed 30 min after initiation of illumination by AL. 

The changes in the O_2_ exchange rate by protoplasts, which were measured using an Oxygraph Plus System (Hansatech Instruments Ltd., Norfolk, UK), were used for the estimation of the responses of photosynthesis (under illumination by blue actinic light, 460 nm, 240 µmol m^−2^ s^−1^) and respiration (under dark conditions). The temperature of the suspension during the measurement was constant (25 °C); the circulation of a large volume of water around the cuvette from a container with a controlled temperature was used for maintaining temperature. Two equal samples of protoplasts (each of them included 1 mL of incubation medium and 0.2 mL of protoplasts suspension) were prepared before measurement. We injected 20 µL of OV (final concentration was 0.25 mM) for the experimental sample and 20 µL of standard solution (1 mM KCl, 0.5 mM CaCl_2_, and 0.1 mM NaCl) for the control sample after 6 min of measurement. The following changes in the O_2_ exchange rate by protoplasts were measured for 9 min. The final response was calculated as the difference between the experimental and control rates of the O_2_ release/consumption for each experiment. Using the difference decreased the experimental error. Increases in O_2_ exchange rate indicated the increase in O_2_ release (under light conditions) or decrease in O_2_ consumption (under dark conditions). The decrease in O_2_ exchange rate indicated the decrease in O_2_ release (under light conditions) or the increase in O_2_ consumption (under dark conditions). 

### 4.6. Statistics

Different seedlings were used for each experiment. Quantities of repetitions are shown in the figures. Mean values, standard errors, representative records, scatter plots, and Pearson correlation coefficients are presented. The significance of differences was estimated using the Student’s *t*-test. The Kolmogorov–Smirnov test of normality was preliminarily used, which showed that the data distribution did not differ significantly from a normal distribution.

## 5. Conclusions

Our work demonstrated the participation of variation potential-related decreases in the activity of plasma membrane H^+^-ATPase in the induction of photosynthetic and respiration changes. The following points support this conclusion: First, preliminary modification of H^+^-ATPase activity (preliminary sodium orthovanadate and fusicoccin treatments) could influence the parameters of the variation potential and the responses of photosynthesis and respiration. Its inactivation decreased the amplitude of the electrical signals and the magnitudes of the photosynthetic and respiratory changes. In contrast, its activation increased the magnitude of VP-induced changes in photosynthetic CO_2_ assimilation. Second, the variation potential amplitudes and magnitudes of the photosynthetic and respiratory responses were linearly related to the magnitudes of the metabolic component of the resting potential, which showed the activity of H^+^-ATPase. Third, injection of a H^+^-ATPase inhibitor (sodium orthovanadate) into a suspension of pea protoplasts induced photosynthetic and respiratory changes that were similar to the changes induced by the variation potential.

## Supplementary Materials

The following are available online at https://www.mdpi.com/2223-7747/9/11/1585/s1, Figure S1: Linear correlation coefficients between amplitudes of variation potentials and local burning-induced changes in CO_2_ assimilation (ΔA_CO2_), quantum yields of photosystem I (ΔΦ_PSI_) and II (ΔΦ_PSII_), and non-photochemical quenching of chlorophyll fluorescence (ΔNPQ), Figure S2: Example of local burning-induced changes in transpiration rate (E) and the investigated photosynthetic parameters under control conditions (a) and linear correlation coefficients of the magnitudes of the photosynthetic responses to the magnitudes of changes in E (b), Figure S3: Linear correlation coefficients between amplitudes of the variation potentials and local-burning-induced changes in respiration (ΔR_CO2_), Figure S4: Example of a change in the membrane potential induced by 5 mM sodium orthovanadate (OV) and measurement of the metabolic component of the resting potential.
