# Supplementary material for: Inactivation of H+-ATPase Participates in the Influence of Variation Potential on Photosynthesis and Respiration in Peas
Source: Plants (Basel). 2020 Nov 16;9(11):1585. doi: 10.3390/plants9111585 (PMC7697462; doi:10.3390/plants9111585)
Supplement: Supplementary file 1 [file plants-09-01585-s001.zip › Fig. S2.pdf]

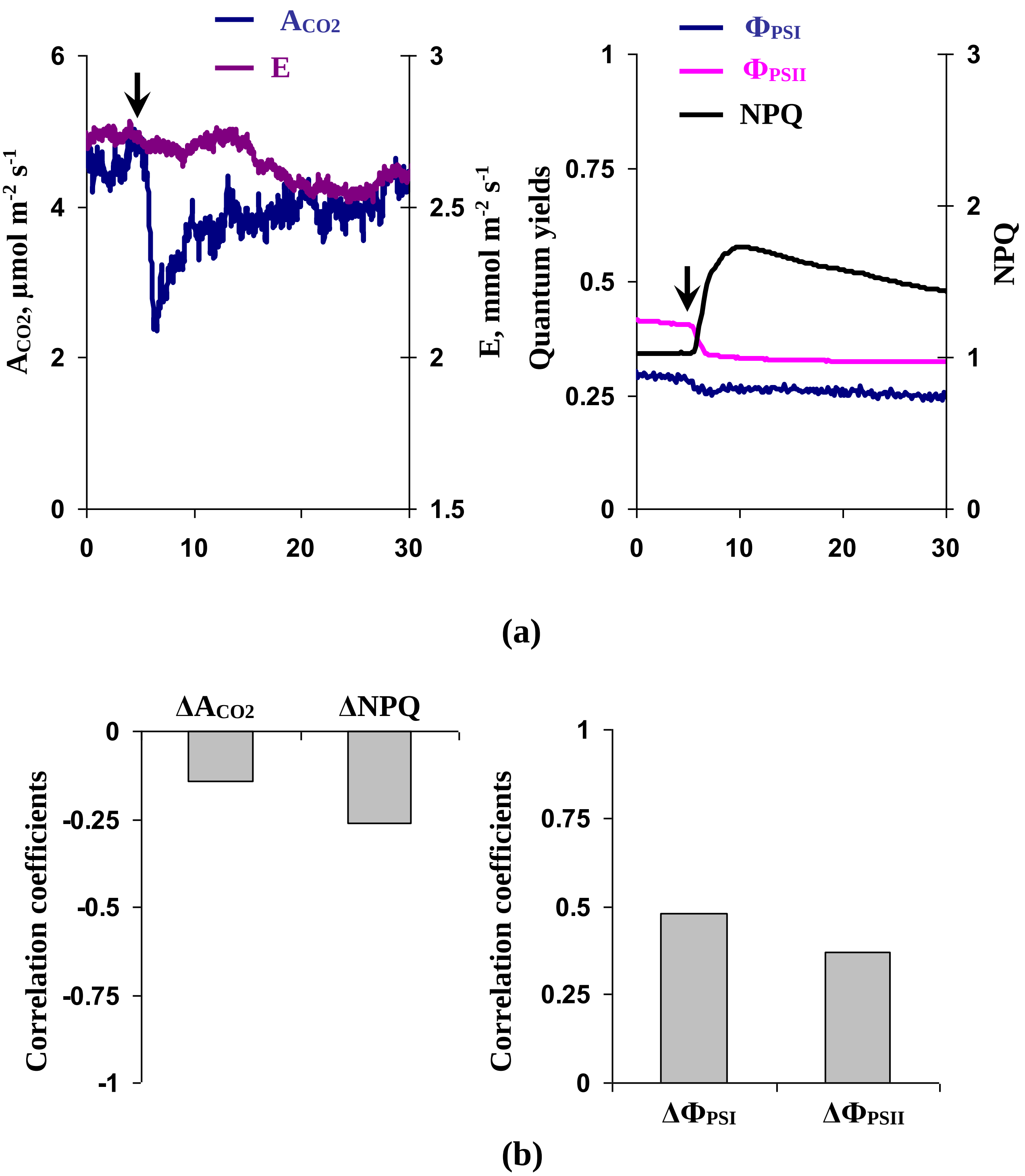

Figure S2. Example of local burning-induced changes in transpiration rate (E) and investigated photosynthetic parameters under control conditions **(a)** and linear correlation coefficients of magnitudes of photosynthetic responses to magnitudes of changes in E **(b)**. Arrow marked the local burning of the first mature leaf (flame, 2-3 s). Correlation coefficients were calculated on basis of all experimental variants for investigation of local burning influence on photosynthesis, including pea seedlings under control conditions, after treatment by 0.5 mM sodium orthovanadate and after treatment by 1 µM fusicoccin (*n*=16). Significant correlation coefficients were absent.
