# Supplementary material for: Inactivation of H+-ATPase Participates in the Influence of Variation Potential on Photosynthesis and Respiration in Peas
Source: Plants (Basel). 2020 Nov 16;9(11):1585. doi: 10.3390/plants9111585 (PMC7697462; doi:10.3390/plants9111585)
Supplement: Supplementary file 1 [file plants-09-01585-s001.zip › Fig. S3.pdf]

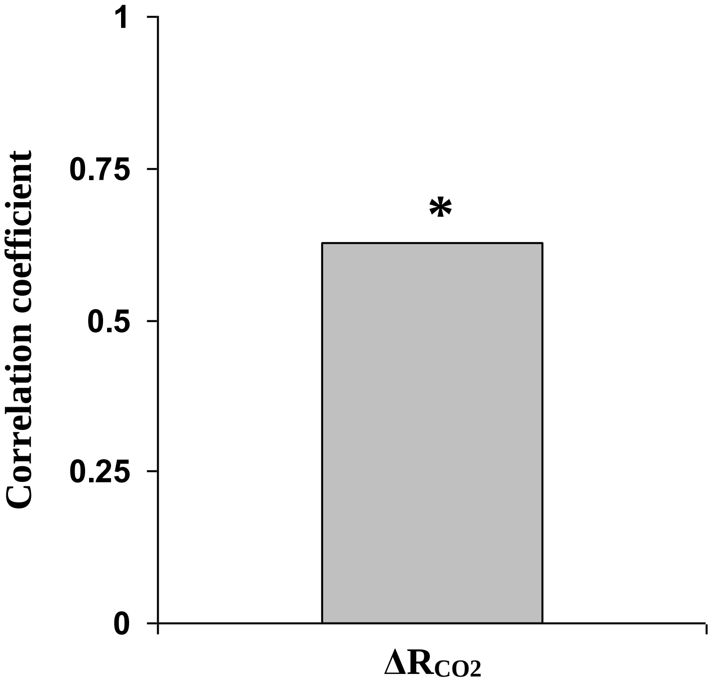

Figure S3. Linear correlation coefficients between amplitudes of variation potentials and local burning-induced changes in respiration (ΔR_{CO2}). Correlation coefficients were calculated on basis of all experimental variants for investigation of local burning influence on respiration, including pea seedlings under control conditions, after treatment by 0.5 mM sodium orthovanadate and after treatment by 1 μM fusicoccin (*n*=16). *, correlation coefficient was significant ($p<0.05$).
