# Supplementary material for: Inactivation of H+-ATPase Participates in the Influence of Variation Potential on Photosynthesis and Respiration in Peas
Source: Plants (Basel). 2020 Nov 16;9(11):1585. doi: 10.3390/plants9111585 (PMC7697462; doi:10.3390/plants9111585)
Supplement: Supplementary file 1 [file plants-09-01585-s001.zip › Fig. S4.pdf]

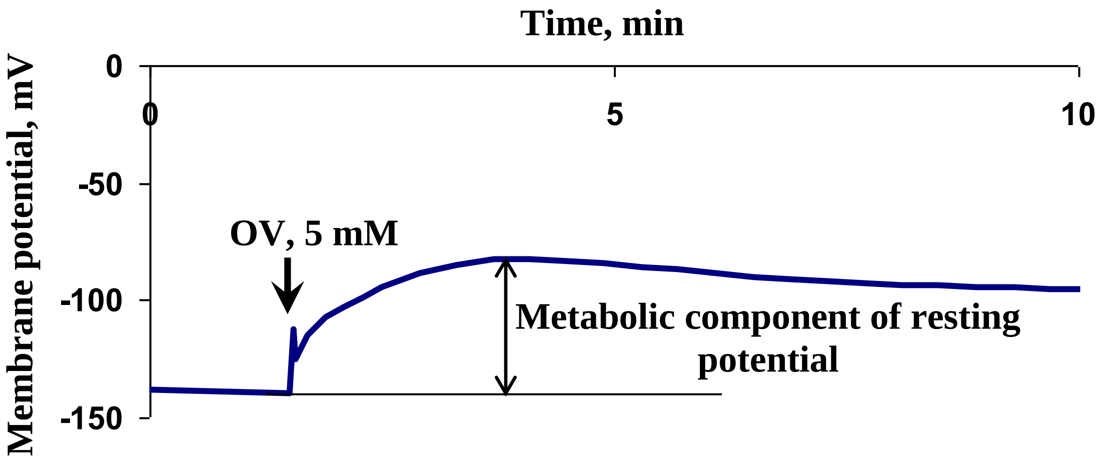

Figure S4. Example of change in membrane potential, induced by 5 mM sodium orthovanadate (OV), and measurement of metabolic component of resting potential. The magnitude of the metabolic component, which showed activity of H^{+}-ATPase in the plasma membrane, was estimated as amplitude of short-term changes in membrane potential after action of the high concentration of OV (5 mM). Arrow marked the injection of OV into solution, which surrounded investigated cells.
